# First detection of *European bat lyssavirus* type 2 (EBLV-2) in Norway

**DOI:** 10.1186/s12917-017-1135-z

**Published:** 2017-07-11

**Authors:** Torfinn Moldal, Turid Vikøren, Florence Cliquet, Denise A. Marston, Jeroen van der Kooij, Knut Madslien, Irene Ørpetveit

**Affiliations:** 1 Norwegian Veterinary Institute, Postbox 750, Sentrum, 0106 Oslo, Norway; 2 Nancy OIE/WHO/EU Laboratory for Rabies and Wildlife, French Agency for Food, Environmental and Occupational Health & Safety, CS 40009, 54220 Malzéville, France; 3 Animal and Plant Health Agency, New Haw, Addlestone, Surrey KT15 3NB UK; 4 Norwegian Zoological Society’s Bat Care Centre, Rudsteinveien 67, 1480 Slattum, Norway

**Keywords:** Rabies, Daubenton’s bat (Myotis daubentonii), European bat lyssavirus type 2 (EBLV-2), fluorescent antibody test (FAT), polymerase chain reaction (PCR), rabies tissue culture infection test (RTCIT)

## Abstract

**Background:**

In Europe, bat rabies is primarily attributed to *European bat lyssavirus* type 1 (EBLV-1) and *European bat lyssavirus* type 2 (EBLV-2) which are both strongly host-specific. Approximately thirty cases of infection with EBLV-2 in Daubenton’s bats (*Myotis daubentonii*) and pond bats (*M. dasycneme*) have been reported. Two human cases of rabies caused by EBLV-2 have also been confirmed during the last thirty years, while natural spill-over to other non-flying mammals has never been reported. Rabies has never been diagnosed in mainland Norway previously.

**Case presentation:**

In late September 2015, a subadult male Daubenton’s bat was found in a poor condition 800 m above sea level in the southern part of Norway. The bat was brought to the national Bat Care Centre where it eventually displayed signs of neurological disease and died after two days. EBLV-2 was detected in brain tissues by polymerase chain reaction (PCR) followed by sequencing of a part of the nucleoprotein gene, and lyssavirus was isolated in neuroblastoma cells.

**Conclusions:**

The detection of EBLV-2 in a bat in Norway broadens the knowledge on the occurrence of this zoonotic agent. Since Norway is considered free of rabies, adequate information to the general public regarding the possibility of human cases of bat-associated rabies should be given. No extensive surveillance of lyssavirus infections in bats has been conducted in the country, and a passive surveillance network to assess rabies prevalence and bat epidemiology is highly desired.

## Background

Rabies is a fatal zoonotic neurological disease caused by RNA viruses belonging to the genus *Lyssavirus* in the family *Rhabdoviridae*. *Rabies virus* (RABV) is the prototype of the genus and causes approximately 59,000 deaths in humans yearly [1]. Red fox (*Vulpes vulpes*) is the main reservoir for RABV in Europe, where the disease has been eliminated in many member states of the European Union due to oral vaccination programs [2]. Rabies in non-flying mammals has never been diagnosed in mainland Norway [3]. However, in the Svalbard archipelago, which is under Norwegian jurisdiction, several occasional detections and two rabies outbreaks during the last 35 years have been reported and are related to arctic strains of RABV in Arctic fox (*Vulpes lagopus*), Svalbard reindeer (*Rangifer tarandus platyrhyncus*) and one ringed seal (*Pusa hispida*) [4–6].

The genus Lyssavirus consists of 14 recognized species [7], and all but two have been isolated from bats [8]. The different species display distinct features regarding geographical distribution and host specificity. In Europe, bat rabies is primarily attributed to *European bat lyssavirus* type 1 (EBLV-1) and *European bat lyssavirus* type 2 (EBLV-2) [9]. Most reported bat rabies cases are caused by EBLV-1 which is almost exclusively found in serotine bats (*Eptesicus serotinus*) and Isabelline serotine bats (*E. isabellinus*), while EBLV-2 is detected mainly in Daubenton’s bats (*Myotis daubentonii*) and to a lesser extent in pond bats (*M. dasycneme*) [9]. Evolutionary studies suggest that EBLV-2 diverged from other lyssaviruses more than 8000 years ago and that the current diversity of EBLV-2 has built up during the last 2000 years [10].

Hitherto, EBLV-2 has been detected in bats in the United Kingdom, the Netherlands, Germany, Switzerland, Finland and Denmark [11–16], while antibodies against EBLV-2 have been found in bats in Sweden [17]. Two cases of human rabies associated with EBLV-2 have been reported in Finland [18] and in Scotland [19]. To date, no cases of natural spill-over to other non-flying mammals have been reported, but EBLV-2 has successfully been transferred to sheep and foxes in experimental studies, even though the susceptibility seems to be low in these species [20, 21].

Among the 12 species of bats regularly reported in Norway, the northern bat *(E. nilssonii*), the soprano pipistrelle (*Pipistrellus pygmaeus*) and the Daubenton’s bat are the most common and widespread species [22–24]. The serotine bat has been recorded once and is not considered as a resident species [25]. The range of the Daubenton’s bat reaches to 63° N in Norway [24]. The results of ringing experiments in other countries suggest that this species does not migrate over long distances [26], which is likely also the case in Norway. The Daubenton’s bat is specialized in hunting insects and spiders, which it mainly catches with its feet from the water surface [27], but it is also found searching for food in forests [24]. Daubenton’s bats seldom occur above the timberline and roost mainly in tree cavities, but also use crevices in bridges and cliffs. Roosts in buildings are very rare [24, 28] and so are contacts with the general public [29].

There is no official surveillance program for lyssavirus in bats in Norway. According to the annals of the Norwegian Veterinary Institute (NVI), a total of 27 bats (swabs from 18 bats sampled alive and brain tissues from nine carcasses) were examined for lyssavirus in the period 1998–2015. Here, we report the first detection of EBLV-2 in Norway.

## Case presentation

### Clinical signs and treatment

On September 29th 2015, a bat was discovered close to a cabin in Valdres in the county of Oppland. The locality is situated just below the timberline at around 800 m above sea level at 60° 59′ N in the southern part of Norway. The landscape is undulating with open spaces and mixed forest dominated by Norway spruce (*Picea abies*) and mountain birch (*Betula pubescens ssp. czerepanovii*), lakes and small meadows*.* The cabin owner discovered the animal clinging to a stone when she removed a tarpaulin. Three days later, the bat was still there, and the cabin owner called the National Bat Helpline, which advised her to bring the bat to the national Bat Care Centre of the Norwegian Zoological Society (NZI). The centre is approved and partly funded by the Norwegian Environmental Agency (NEA) and the Norwegian Food Safety Authority (NFSA) [29].

**Fig. 1:**
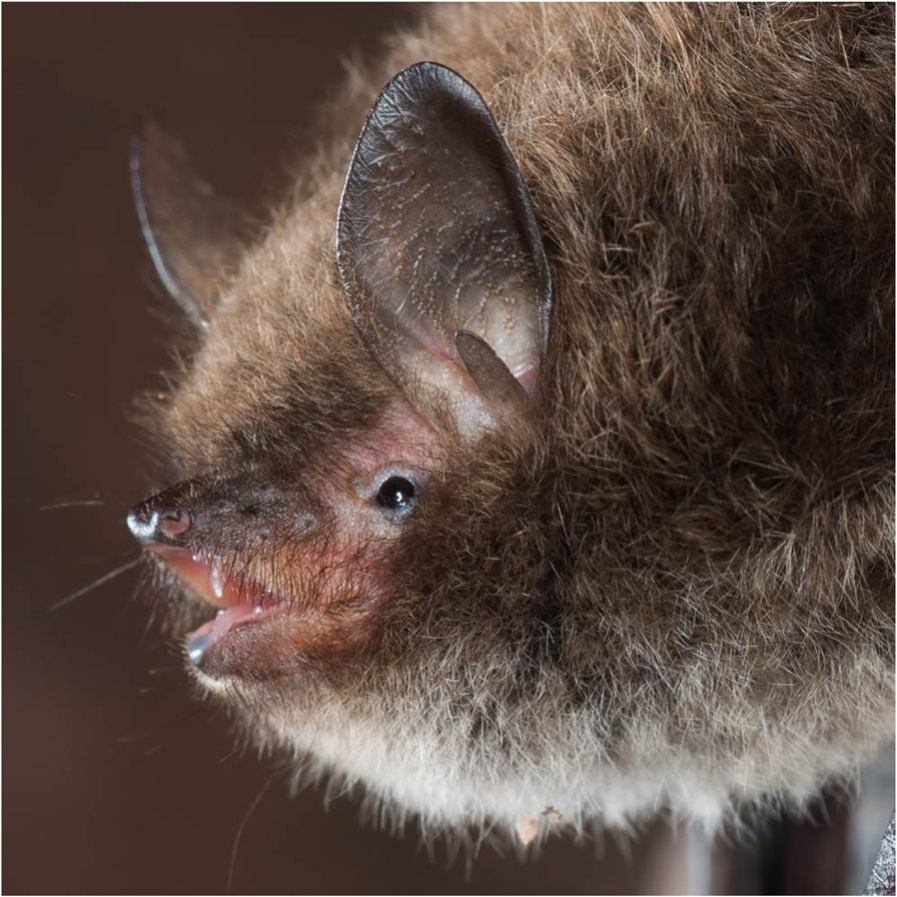
The bat was identified as a subadult male Daubenton’s bat based on external characteristics. Photographer: Jeroen van der Kooij

The bat arrived at the Bat Care Centre the next day, on October 3rd 2016, and was identified as a male Daubenton’s bat based on external characteristics, i.e. short tragus, large feet and the attachment of the wing membrane at the middle of the foot, and as a subadult individual based on the brownish face and the presence of a chin-spot (Fig. 1) [30]. The bat weighed 6.6 g on arrival and had a forearm length of 38.3 mm, hence the body condition index (mass g/forearm mm) was 0.17, which indicated low energy reserves [31]. It was weak, and the tongue was dark red and swollen. It had dark plaque on its teeth, and there was a heavy load of mites of the genus *Spinturnix* on the wing membrane surface. As a safety precaution, the diseased bat was routinely placed in a clean plastic container (35x45x40 cm) separated from other bats in the facility.

The bat drank and ate mealworm (*Tenebrio molitor*) on arrival. It walked around inside the container and on one occasion lunged against the bat carer. After a few hours the bat grew stronger, groomed itself and produced some excrement. The following day, on October 4th 2016, it gradually refused to eat, displayed difficulty when swallowing water and got weaker. The bat was force-fed several times, received fluid subcutaneously and eventually had increased locomotor activity. In the late evening it displayed mild ataxia. The following morning, the bat’s condition deteriorated. The bat walked around, but its locomotion was less agile and it was not able to groom itself. The bat became even weaker towards the evening in spite of treatment with antibiotics and fluid, and when force-fed with water or fluids, it showed signs of vomiting and spread both wings and legs. It also tried to bite, but without force. The bat died in the evening of October 5th 2016 and was subsequently frozen.

### Necropsy, sampling and histology

On October 6th 2015, the bat was submitted to the NVI in Oslo for laboratory examinations on suspicion of rabies. The necropsy revealed that the bat was in a poor condition with no visible body fat. The tongue was dark red and dry. The internal organs were congested with no specific gross findings.

Brain impressions on glass slides were fixed in acetone baths for fluorescent antibody test (FAT), and tissues from different parts of the brain were put on empty tubes and tubes with lysis buffer for rabies tissue culture infection test (RTCIT) and detection of viral RNA by reverse transcription and polymerase chain reaction (RT-PCR), respectively. The remains of the brain and the head including salivary glands, lung, heart, liver, kidney, spleen, gastrointestinal tract and pancreas were fixed in 10% buffered formalin for two weeks, routinely processed and embedded in paraffin before cutting ultrathin sections that were mounted on glass slides and stained with haematoxylin-eosin for histological examination.

Only small pieces of brain tissue were available for histology, and no lesions were detected. In the lungs, multifocal areas of foreign material aspiration with infiltration of inflammatory cells were seen, and a similar small focal inflammation was found in the salivary gland tissue. A few parasitic structures without any associated inflammation were detected in the liver.

### FAT and RTCIT

The laboratory tests used for rabies diagnosis are recommended by WHO and OIE. FAT, in which virus antigens in brain tissues are detected, is the gold standard for diagnosing rabies [32]. Fixed brain impressions were stained with FITC Anti-Rabies Monoclonal Globulin (Fujirebio Diagnostics Inc.) according to the manufacturer’s instructions. A brain impression from a rabid cow served as a positive control, while a brain impression from a healthy fox served as a negative control. All slides were investigated in a fluorescence microscope (Leitz) under ultraviolet light. Virus antigen was detected in the positive control, but not in the negative control or any of the brain impressions from the bat.

The detection of infectious particles in brain homogenates was performed by RTCIT on neuroblastoma cells with an incubation period of 48 h at 37 °C as previously described [33]. The staining was performed using a rabies anti-nucleocapsid FITC-conjugated antibody (BioRad). The brain tissues from the bat were found positive for the presence of infectious virus by RTCIT.

### Nucleic acid extraction, RT-PCR and partial sequencing of the nucleoprotein gene

Total nucleic acids were extracted using the NucliSens® easyMAG™ (bioMerieux Inc.) according to the manufacturer’s instructions for the off-board protocol. Nucleic acids were eluted in 55 μl buffer. Primers targeting the nucleoprotein gene were applied in a two-step RT-PCR. RT was performed with Invitrogen SuperScript® III Reverse Transcriptase (Thermo Fisher Scientific) with the primer LYSSA-NMA (5′-ATGTAACACCYCTACAATG-‘3) that is modified from primer JW12 in [34].

PCR was performed with Qiagen HotStarTaq® DNA Polymerase (Qiagen) according to the manufacturer’s instructions. The forward primer, LYSSA-NMB (5′-ATGTAACACCYCTACAATGGA-‘3) was used in two separate PCR reactions with either LYSSA-NGA (5’-TGACTCCAGTTRGCRCACAT-‘3) or LYSSA-NGC (5’-GGGTACTTGTACTCATAYTGRTC-‘3) as reverse primers, yielding amplicons of 612 bp and 108 bp respectively. The primers are modified from the primers JW12, JW6 and SB1 in [34] respectively. Amplicons were separated on 1% agarose gel at 90 V for 90 min and visualized by DNA Gel Loading Dye (Thermo Fisher Scientific).

The PCR products yielding bands of the expected size in the agarose gel electrophoresis were further analysed by DNA sequencing. Following enzymatic PCR clean up with illustra™ ExoStar™ (GE Healthcare Life Sciences), DNA sequencing was performed using the BigDye® Terminator v3.1 Cycle Sequencing Kit (Thermo Fisher Scientific) according to the manufacturer’s instructions. The products were analysed in an ABI PRISM® 3100 Genetic Analyzer (Thermo Fisher Scientific) according to the manufacturer’s instructions and with Sequencher® version 5.3 sequence analysis software (Gene Codes Corporation).

**Fig. 2:**
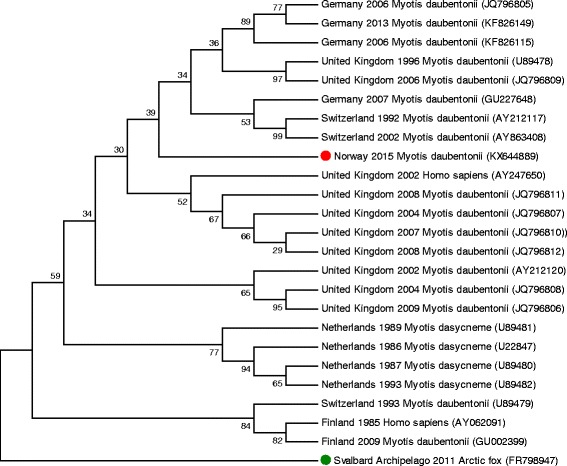
Phylogenetic relationship between the Norwegian EBLV-2 sequence  and other EBLV-2 sequences based on 391 nucleotides from the nucleoprotein gene. The tree was constructed with the Maximum Likelihood algorithm with 1000 bootstrap replicates and the Tamura-Nei substitution model. The number at each branch of the phylogenetic tree represents the likelihood in percentage that the sequences cluster together. RABV from an Arctic fox is used as outgroup . The country and year of collection, host species and GenBank accession number for sequences are given

A sequence with 567 nucleotides (GenBank accession number KX644889) was obtained with the primers LYSSA-NMB and LYSSA-NGA. BLAST search against the GenBank database showed 94–96% identity with EBLV-2 (Table 1). The sequence was aligned with sequences for EBLV-2 published in GenBank to evaluate the genetic diversity and the relationship to other bat-associated lyssaviruses, and phylogenetic trees were constructed with MEGA 6 based on an alignment of 391 nucleotides applying the Maximum Likelihood and Neighbor-Joining algorithms with 1000 bootstrap replicates and different substitution models (Fig. 2) [35]. The trees showed similar results, and although the node is not strongly supported, the results indicate that the sequence from the Norwegian virus did not cluster closely with any other published sequences of EBLV-2.

**Table 1:** The site and year of collection, host species and GenBank accession number for sequences for a part of the nucleoprotein gene of *European bat lyssavirus* 2 used to generate a phylogenetic tree

Place	Country	Year	Host species	GenBank accession number	Reference(s)
Helsinki	Finland	1985	*Homo sapiens*	AY062091	[18, 61]
Wommels	Netherlands	1986	*Myotis dasycneme*	U22847	[12, 62]
Tjerkwerd	Netherlands	1987	*Myotis dasycneme*	U89480	[12, 62]
Andijk	Netherlands	1989	*Myotis dasycneme*	U89481	[12, 62]
Plaffeien	Switzerland	1992	*Myotis daubentonii*	AY212117	[14, 46]
Roden	Netherlands	1993	*Myotis dasycneme*	U89482	[12, 62]
Versoix	Switzerland	1993	*Myotis daubentonii*	U89479	[62]
Sussex	United Kingdom	1996	*Myotis daubentonii*	U89478	[11, 62]
Lancashire	United Kingdom	2002	*Myotis daubentonii*	AY212120	[46]
Angus	United Kingdom	2002	*Homo sapiens*	AY247650	[19]
Geneva	Switzerland	2002	*Myotis daubentonii*	AY863408	[63]
Surrey	United Kingdom	2004	*Myotis daubentonii*	JQ796807	[9, 47]
Lancashire	United Kingdom	2004	*Myotis daubentonii*	JQ796808	[9, 64]
Oxfordshire	United Kingdom	2006	*Myotis daubentonii*	JQ796809	[9, 48]
Magdeburg	Germany	2006	*Myotis daubentonii*	JQ796805	[9, 50]
Schwansee	Germany	2006	*Myotis daubentonii*	KF826115	[65]
Bad Buchau	Germany	2007	*Myotis daubentonii*	GU227648	[13]
Shropshire	United Kingdom	2007	*Myotis daubentonii*	JQ796810	[9, 49]
Surrey	United Kingdom	2008	*Myotis daubentonii*	JQ796811	[9]
Shropshire	United Kingdom	2008	*Myotis daubentonii*	JQ796812	[9, 66]
West Lothian	United Kingdom	2009	*Myotis daubentonii*	JQ796806	[9, 67]
Turku	Finland	2009	*Myotis daubentonii*	GU002399	[15]
Gießen	Germany	2013	*Myotis daubentonii*	KF826149	[65]
Valdres	Norway	2015	*Myotis daubentonii*	KX644889	This study

### Whole genome sequencing

Total RNA was depleted of host genomic DNA (gDNA) and ribosomal RNA (rRNA) following methods described previously [36, 37]. Briefly, gDNA was depleted using the on-column DNase digestion protocol in RNeasy plus mini kit (Qiagen) following manufacturer’s instructions, eluting in 30 μl molecular grade water. Subsequently, rRNA was depleted, using Terminator 5′-phosphate-dependent exonuclease (Epicentre Biotechnologies). Briefly, 30 μl of gDNA depleted RNA was mixed with 3 μl of Buffer A, 0.5 μl of RNAsin Ribonuclease inhibitor (20–40 U/μl) and incubated at 30 °C for 60 min. The depleted RNA was purified to remove the enzyme using the RNeasy plus mini kit as above, without the DNase digestion, eluting in 30 μl of molecular grade water. Double stranded cDNA (ds-cDNA) was synthesised using random hexamers, and a cDNA synthesis kit (Roche) following manufacturer’s instructions. The resulting ds-cDNA was purified using AMPure XP magnetic beads (Beckman Coulter), quantified using Quantifluor (Promega) and approximately 1 ng of the ds-cDNA library was used in a ‘tagmentation’ reaction mix using a Nextera XT DNA sample preparation kit (Illumina) following manufacturer’s instructions – without the bead normalization step. The DNA library was quantified using Quantifluor (Promega) and sequenced as 2 × 150 bp paired-end reads on an Illumina MiSeq platform.

Short reads were mapped to the most genetically related full length EBLV-2 genome available (Germany 2012 – GenBank accession number KY688149). Reads were mapped using the Burrow-Wheeler Aligner (BWA version 0.7.5a–r405) [38] and visualized in Tablet [39]. A modified SAMtools/vcfutils [40] script was used to generate an intermediate consensus sequence in which any indels and SNPs relative to the original reference sequence were appropriately called. The intermediate consensus sequence was used as the reference for four subsequent iterations of mapping and consensus calling, described previously [41].

A total of 38,288 EBLV-2 specific reads were mapped from 5,839,126 total reads (0.66%). The average depth of coverage was 373.6 with a maximum depth of coverage of 1900. Sequencing resulted in complete genomic coverage, apart from the first 9 nucleotides. However, the lyssavirus genomic ends are highly conserved between species and palindromic [42, 43]. Therefore the first 9 nucleotides of the 3′ UTR sequence was deduced using the 5′ end sequence and matched 100% with other EBLV-2 genomes available. The total genomic length is 11,928 nucleotides (Table 2), conforming to other EBLV-2 genome sequences (11,924–30), where all regions are identical in length except for the non-coding regions of M-G and G-L. The complete genome sequence was submitted to GenBank (accession number KY688154).

**Table 2:** Length of coding (bold) and non-coding regions of EBLV-2 in nucleotides (nts)

Region	Length (nts)
3′ UTR	70
**N protein**	1356
N-P	101
**P protein**	894
P-M	88
**M protein**	609
M-G	210
**G protein**	1575
G-L	510
**L protein**	6384
5′ UTR	131
**Total**	**11,928**

## Discussion and conclusions

Here, we report the first detection of EBLV-2 in Norway. The detection of EBLV-2 in a Daubenton’s bat is in accordance with previous studies that have revealed that EBLV-2 is mainly found in this species [9]. As the bat was a subadult and ringing experiments suggest that this species does not migrate over long distances [26], it can be assumed that the bat was native to the area where it was found and that EBLV-2 is present in Norway. The latter is supported by the phylogenetic analyses, which indicate that the Norwegian isolate differs from other published isolates, further suggesting that the Norwegian isolate may have evolved separately from a common European ancestor [10]. Very few bats have been examined for rabies in Norway, and we have no knowledge of the prevalence and epidemiology of EBLV. In neighbouring countries, EBLV-2 was detected in diseased Daubenton’s bats in Finland in 2009 and 2016 [15, 44], while active surveillance has revealed viral RNA from mouth swabs from Daubenton’s bat in Denmark and seropositive Daubenton’s bats in both Sweden and Finland [16, 17, 45].

The FAT performed on brain impressions at the NVI was negative, and unfortunately, due to insufficient material, it could not be repeated at the Nancy OIE/WHO/EU Laboratory for Rabies and Wildlife (EURL). However, the EURL confirmed the presence of lyssavirus in the RTCIT. The negative result for FAT does not concur with findings in other Daubenton’s bats naturally infected with EBLV-2 [11, 13–15, 44, 46–51]. The annual proficiency tests for rabies diagnosis techniques organized by the EURL have demonstrated the difficulty for laboratories to reliably detect EBLV strains when using the FAT [52], with results depending on the rabies virus antibody conjugate and even the batch used [53]. However, the same batch used in the FAT for the present case has successfully been applied at the NVI for detection of this virus species in two proficiency tests (unpublished results). A polyclonal antibody was used to confirm the presence of lyssavirus in the RTCIT, and a difference between the antibodies regarding the ability to detect the current virus cannot be excluded.

The aspiration pneumonia might have been caused by a dysfunctional swallow reflex or as a result of the force-feeding. The bat had a low state of nutrition, displayed several signs of weakness and, in the end, an inability to fly. This could be caused by the EBLV-2 infection or due to general weakness as a consequence of either shortage of feed at the high altitude or inexperience in hunting since being a subadult. Also, a poor body condition could have made the bat more susceptible for EBLV-2 infection. Weight loss, the inability to fly and death within 14 days followed by detection of viral antigen in brain tissue is reported in Daubenton’s bats after intracerebral inoculation with EBLV-2 [54]. In that study the bats were fed ad libitum, so the weight loss could not be related to a decrease in hunting success. During a natural EBLV-2 infection, the course of the disease is probably longer than that found in the referred experiment, and thus the ability to hunt and eat could deteriorate over some time span, resulting in gradual loss of body condition. Poor body condition in bats can also be influenced by heavy parasite loads [55]. Other possible signs of the disease, like the inability to fly, were registered only up to 48 h before death in the experiment [54]. Most of the bats which were inoculated either intramuscularly or subdermally survived the study period of 123 days [54]. These findings highlight that there is a high intra-species barrier in transmission of EBLV-2 and that the incubation period under natural conditions is probably several weeks to months.

Two human cases of rabies caused by EBLV-2 have been reported, in Finland in 1985 and in Scotland in 2002 [18, 19]. Both persons were in close contact with bats over time and had been bitten several times without any history of immunization prior to exposure or post-exposure prophylaxis. Spill-over to other non-flying mammals under natural conditions has not been reported, and the detection of EBLV-2 does not pose an immediate risk to humans as Daubenton’s bats are rarely in contact with the public [29]. However, the possibility of human cases of bat-associated rabies acquired in Norway, which is considered free of rabies, cannot be neglected; hence adequate information should be given to the general public as well as to people and professionals who come into contact with bats.

All bat species in Norway are strictly protected under both national laws and international commitments to preserve bats [56, 57]. Culling bats for lyssavirus testing is therefore not a feasible strategy. Passive surveillance by testing bats found sick or dead is the most appropriate way of assessing the incidence of rabies as compared with active surveillance by testing swabs and/or sera from live bats from natural populations [58, 59]. Passive surveillance of bat rabies is therefore strongly recommended by international organizations [58, 60].

Monitoring of EBLV in bats has so far not been prioritized by the Norwegian authorities, probably due to few human cases of bat-associated rabies being reported in Europe. According to the annals of the NVI, only seven carcasses of bats including the EBLV-2 positive case were examined during the last three years. In 2014 and 2015, the NVI in collaboration with the NZS collected and tested nasopharyngeal and cloacal swabs from totally 16 free-ranging Norwegian bats for lyssaviruses with a negative result (unpublished data). The experience gained in this pilot study and from surveillance in other countries is important for future monitoring of bat rabies in Norway.

The authors believe that testing bats found sick or dead should get priority through a national rabies surveillance network in line with the recent recommendations of EUROBATS and EFSA [58, 60]. Additionally, an active surveillance program, over a limited number of years and in defined areas to obtain baseline data on the prevalence of bat rabies in Norway, is also recommended. Finally, we emphasize the importance of good cooperation between bat biologists and laboratory workers to ensure access to representative samples of good quality.

## Abbreviations

EBLV-1: *European bat lyssavirus* type 1
EBLV-2: *European bat lyssavirus* type 2
EURL: Nancy OIE/WHO/EU Laboratory for Rabies and Wildlife
FAT: Fluorescent antibody test
gDNA: Genomic DNA
NEA: Norwegian Environmental Agency
NFSA: Norwegian Food Safety Authority
NVI: Norwegian Veterinary Institute
NZI: Norwegian Zoological Society
PCR: Polymerase chain reaction
RABV: *Rabies virus*
rRNA: Ribosomal RNA
RT: Reverse transcription
RTCIT: Rabies tissue culture infection test
